# Saunders’ Pocket Medical Lexicon

**Published:** 1891-01

**Authors:** 


SAUNDERS' POCKET MEDICAL LEXICON. By John M. Keating, M. D., and Henry Hamilton. Published by W. B. Saunders. Philadelphia, 1890, price, Cloth 75 cents ; Morocco tucks, $1.

This volume though small, contains much information which the adept as well as the tyro will find useful, and from its size recommends itself to the student, as he can cary it in his pocket with little discomfort, thus having ever by him a friend most willing to enlighten.

